# Editorial: Applied neuroimaging for the diagnosis and prognosis of cerebrovascular disease

**DOI:** 10.3389/fneur.2025.1714644

**Published:** 2025-10-15

**Authors:** Mingming Lu, Jieqiong Wang, Jianming Cai

**Affiliations:** ^1^Department of Radiology, Pingjin Hospital, Characteristic Medical Center of Chinese People's Armed Police Force, Tianjin, China; ^2^Department of Neurological Sciences, University of Nebraska Medical Center, Omaha, NE, United States; ^3^Department of Radiology, The Fifth Medical Center of Chinese People's Liberation Army of China (PLA) General Hospital, Beijing, China

**Keywords:** neuroimaging, cerebrovascular diseases, diagnosis, prognosis, stroke

Neuroimaging plays a crucial role in the diagnosis and prognosis of cerebrovascular diseases. By using advanced imaging techniques, neuroimaging can reveal the anatomical structure and function of the brain, providing important evidence for diagnosing and treating these conditions. This Research Topic summarizes 14 recent original research studies that explore the application of advanced imaging techniques to evaluate and predict the outcomes of key neurological disorders, including intracranial arterial disease (ICAD)-related ischemic stroke, acute ischemic stroke (AIS) post-mechanical thrombectomy, transient ischemic attack (TIA), aneurysmal subarachnoid hemorrhage (aSAH), and heart failure. The studies highlight how imaging modalities such as computed tomography (CT), multimodal magnetic resonance imaging (MRI), high-resolution vessel wall imaging (HR-VWI), and arterial spin labeling (ASL) can provide critical insights into disease pathophysiology, temporal dynamics, and prognosis, offering valuable tools for clinical decision-making.

CT and CTA are foundational in vascular neurological assessments due to their speed and accessibility. CTA was used in the ANTIQUE study (Pakizer et al.) to classify carotid plaque calcification in extracranial carotid artery disease into spotty (< 3 mm) and large (>3 mm) types. The authors found that spotty calcification correlated with male sex and heavy smoking (*p* = 0.014), while large calcification was associated with older age, coronary heart disease, and atrial fibrillation (*p* = 0.025). In acute stroke, CTA assesses stenosis severity and plaque morphology. Chen et al. showed that acute stroke patients have higher systolic blood pressure, thicker plaques, and more severe stenosis on CTA compared to non-acute patients. CTA—derived perivascular fat density (PFD) was found to be a strong predictor of acute ischemia, with symptomatic-side PFD outperforming contralateral PFD. Kim, Kim et al. retrospectively analyzed 114 aSAH patients to explore the clinical significance of mastoid effusion (ME)—defined as opacification/air-fluid levels in the mastoid air cells on CT/MRI within 14 days of aSAH. Multivariate analysis showed that ME was independently associated with tracheostomy, radiologic vasospasm, higher APACHE II scores, and poor outcomes (90-day mRS > 2, OR = 4.289, *p* = 0.041). Wei et al. investigated the prognostic value of the gray-to-white matter ratio (GWR) on cranial CT scans in 86 heat stroke patients (derivation cohort) and 42 patients (validation cohort), respectively. All GWR parameters were lower in the poor outcome group. GWR basal ganglia showed the highest sensitivity (80.95%) at 90.77% specificity (cut-off = 1.21) in the derivation cohort, with an AUC of 0.852. Combining GWR basal ganglia with qSOFA (quick Sequential Organ Failure Assessment) significantly improved sensitivity and the AUC (0.958 vs. 0.852 for GWR alone, *p* = 0.034). This study establishes GWR as an objective, early predictor of poor neurological outcomes in heat stroke.

Conventional magnetic resonance imaging (MRI) sequences, such as T1— weighted, T2—weighted, FLAIR, and diffusion-weighted imaging (DWI), provide detailed insights into brain structure and function. In heart failure with preserved ejection fraction (HFpEF), Yu et al. used voxel-based morphometry (VBM) on 3D T1—weighted images and found reduced gray matter volume (GMV) in the bilateral cerebellar hemispheres, right posterior cingulate gyrus, and right inferior frontal gyrus in HFpEF patients. These GMV reductions correlated negatively with NT-proBNP levels and MoCA scores, linking cardiac dysfunction to cognitive impairment and structural brain changes. For AIS prognosis, Pei et al. integrated multimodal MRI with radiomics and deep learning. The authors extracted 1,197 radiomic features, selected 16 via LASSO regression, and developed a CRD (Clinic-Radiomics-Deep Learning) model. The CRD model achieved an AUC of 0.908 in the validation cohort, outperforming clinical (AUC = 0.874) and radiomics (AUC = 0.805) models alone. This highlights the role of MRI in capturing subtle pathological features for personalized prognosis. Almeida et al. explored the link between PCS symptoms (e.g., chronic fatigue, headaches) and T2-hyperintense white matter lesions using MRI in 96 Swiss patients. The majority of patients were women (73%, average age 46), with a high prevalence of chronic fatigue (90%), headaches (57%), and sleep disorders (51%). Brain MRIs showed lesions in 72% of patients, while spinal MRIs showed lesions in 16% of subjects. However, there was no significant correlation between lesions and fatigue (*p* = 0.815) or headaches (*p* = 0.178). This suggests that T2-hyperintense lesions may not be the cause of these PCS symptoms.

This Research Topic included two studies evaluating the application of high-resolution vessel wall imaging (HR-VWI) in treating cerebrovascular diseases. Kang et al. conducted a longitudinal HR-VWI study on 208 ICAD patients and found that arterial dissection led to faster stenosis reduction and an enhancing proportion decline compared to atherosclerosis. Atherosclerosis, however, showed a decreasing enhancement ratio. Thus, HR-VWI aids in monitoring disease progression and guiding treatment. Bao et al. reported a rare case of right type II persistent proatlantal intersegmental artery (PPIA) dissection that caused embolic showers (ES) in a 53-year-old man with hypoplasia of the left vertebral artery. DSA and HRMR-VWI identified aneurysmal dilation in the PPIA's false lumen, and a risk of thrombus dislodgement. Pipeline embolization device (PED)-assisted angioplasty resolved the issue, with no recurrent strokes post-operatively, highlighting the value of PEDs in managing rare vascular variants.

Functional neuroimaging techniques, such as arterial spin labeling (ASL), resting-state functional MRI (rs-fMRI), and electroencephalography (EEG), provide more information for evaluating the diagnosis and prognosis of cerebrovascular disease. Zeng et al. used dual post-label delays (PLD: 1,525 and 2,525 ms) in TIA patients with large artery stenosis/occlusion. FLAIR vascular hyperintensity (FVH)—positive patients had lower CBF on the affected side at both PLDs and a smaller CBF increase than FVH-negative patients. ΔCBF correlated negatively with ABCD2 scores, establishing FVH as a marker for hemodynamic impairment. In OLE, Kim, Ah et al. used diffusion tensor imaging (DTI)—derived peak width of skeletonized mean diffusivity (PSMD), an EEG-related marker. OLE patients had higher PSMD than controls, indicating small vessel disease-related white matter damage. PSMD also correlated with age, positioning it as a novel marker for OLE-associated microvascular changes. Huai et al. assessed the effect of enriched rehabilitation (ER) on post-stroke cognitive impairment (PSCI). Forty PSCI patients were randomly divided into a conventional medical rehabilitation (CMR) group and an ER group, along with 20 healthy controls. The functional connectivity (FC) analysis in the ER group revealed strengthened positive FC between the right dorsolateral prefrontal cortex (DLPFC) and the left superior frontal gyrus (SFG) and left anterior cingulate gyrus (ACG), and decreased FC between the right DLPFC and the right superior temporal gyrus (STG) and right precentral gyrus. ER intervention is more effective than conventional rehabilitation, possibly by reshaping brain functional connectivity. EEG is vital for assessing neural activity and epilepsy. Liao et al. conducted a bibliometric analysis showing a surge in EEG stroke research post-2017, with focus areas including seizure detection, consciousness assessment, and brain-computer interfaces (BCI). Additionally, a bibliometric analysis was conducted by Lou et al. to examine the focal areas of research in the early diagnosis of stroke through machine learning identification of magnetic resonance imaging characteristics from 2004 to 2023. The researchers found that the application of machine learning to the early prediction of stroke and to personalized medical plans for patients using neuroimaging characteristics offers significant value.

In conclusion, multimodal neuroimaging collectively enhances the diagnosis, prognosis, and treatment of neurological disorders. Future research should standardize protocols, validate findings in multicenter cohorts, and integrate multimodal data for more precise clinical care.

